# Supplementary material for: Optimal Pair Matching Combined with Machine Learning Predicts a Significant Reduction in Myocardial Infarction Risk in African Americans Following Omega-3 Fatty Acid Supplementation
Source: Nutrients. 2024 Sep 2;16(17):2933. doi: 10.3390/nu16172933 (PMC11397525; doi:10.3390/nu16172933)

Optimal Pair Matching Combined with Machine Learning Predicts a Significant Reduction in Myocardial Infarction Risk in African Americans following Omega-3 Fatty Acid Supplementation

Shudong Sun, et al.

# Online Supplementary Material

**Table S1:** VITAL Study Data Dictionary

| **Variable** | **Variable Name** | **Variable Label** |
|---|---|---|
| Subject ID | Subject_ID | Assigned Study ID |
| Randomization to active Vitamin D | vitdactive | Vitamin D 1-Active,0-Placebo |
| Randomization to active n−3 fatty acids | fishoilactive | Omega-3 Fatty Acids 1-Active,0-Placebo |
| **Demographic Variables** | | |
| Female sex | sex | Sex 1-male,2-female |
| Age (integer years, truncated at 90+) | ageyr | Age at randomization to VITAL study, years |
| Race or ethnic | raceth | Race/ethnic group 1-NonHisp White,2-Black,3-Hispanic,4-Asian,5-Native American or Alaskan,6=Others/unknown |
| **Baseline Variables** | | |
| Body-mass index (kg/m$^2$) | bmi | Body mass index at randomization, kg/m2 |
| Current smoking | currsmk | Current smoking 1-yes,0-no |
| Hypertension treated with medication | htnmed | Hypertension treated with medication 1-yes,0-no |
| Current use of cholesterol-lowering medication | cholmed | Current use of cholesterol-lowering medications 1-yes,0-no |
| Diabetes | diabetes | Baseline diabetes 1-yes,0-no |
| Use of medication for diabetes | diabmed | Medication for diabetes 1-yes,0-no |
| Parental history of myocardial infarction | parhxmi | Parental history of myocardial infarction 1-yes,0-no |
| Fish consumption (>=median of 1.5 servings / week) | fish1_5wk | Total fish intake >=1.5 servings per week 1-yes, 0-no |
| Baseline aspirin use | Aspirin | Baseline Aspirin use 1-yes,0-no |
| Baseline statin use | statins | Baseline statin 1-yes,0-no |
| No. of cardiovascular risk factors (0, 1, 2+) | N_risk_factors | Number of cardiovascular risk factors 0-None,1-One risk factor,2->one risk factor |
| Baseline Vitamin D intake <= 800 IU | VitDIntake | Baseline vitamin D intake 1-none,2-<=800 IU |
| Baseline 25(OH)D level ≥ 20 ng/ml (excluded due to a large proportion of missing values) | VitD20 | Baseline serum 25-hydroxyvitamin D 1->=20, 0-< 20 ng/mL |
| Baseline 25(OH)D level ≥ 31 ng/ml (excluded due to a large proportion of missing values) | VitD31 | Baseline serum 25-hydroxyvitamin D median 1->=31, 0-< 30 ng/mL |

**Table S2:** Demographic Table before Matching (Original Participant Set, excluding Missing Data).

| **Characteristic** | | **Total (N=19319)** | **AfAm (N=3766)** | **NHW (N=15553)** |
|---|---|---|---|---|
| Age | | 66.4±6.9 | 62.4±6.6 | 67.4±6.7 |
| Female | | 9767 (50.6) | 2347 (62.3) | 7420 (47.7) |
| BMI | | 28.0±5.6 | 30.6±6.5 | 27.4±5.2 |
| Current smoking | | 1309 (6.8) | 528 (14.0) | 781 (5.0) |
| Hypertension medication | | 9380 (48.6) | 2446 (64.9) | 6934 (44.6) |
| Cholesterol medication | | 7257 (37.6) | 1200 (31.9) | 6057 (38.9) |
| Diabetes | | 2429 (12.6) | 871 (23.1) | 1558 (10.0) |
| Diabetes medication | | 1880 (9.7) | 675 (17.9) | 1205 (7.7) |
| Parental history of MI | | 3143 (16.3) | 591 (15.7) | 2552 (16.4) |
| Fish consumption (≥1.5/wk) | | 9100 (47.1) | 1890 (50.2) | 7210 (46.4) |
| Aspirin use | | 8816 (45.6) | 1431 (38.0) | 7385 (47.5) |
| Statin use | | 6790 (35.1) | 1114 (29.6) | 5676 (36.5) |
| Vitamin D supplements | | 8594 (44.5) | 1096 (29.1) | 7498 (48.2) |
| CVD risk factors | 0 | 5833 (30.2) | 778 (20.7) | 5055 (32.5) |
| | 1 | 6603 (34.2) | 1315 (34.9) | 5228 (34.0) |
| | >1 | 6883 (35.6) | 1673 (44.4) | 5210 (33.5) |

**Table S3:** Regression Results after Selection of Variables by LASSO with Stroke as the Outcome using AfAm and NHW Participants after Optimal Pair Matching. Includes interaction terms. Std. Errors, p-values and 95% CI for OR are calculated using non-parametric bootstrap and parametric bootstrap. OR: odds ratio; CI: confidence interval. n-3 HUFA supplementation OR (95% CI) for AfAm subgroup and for NHW subgroup.

| Variables | Estimate | OR | Non-parametric Bootstrap | | | Parametric Bootstrap | | |
|---|---|---|---|---|---|---|---|---|
| | | | Std. Error | P-value | 95% CI for OR | Std. Error | P-value | 95% CI for OR |
| (Intercept) | -4.4923 | 0.0112 | 1.9847 | 0.0236 | (0.0002, 0.5476) | 1.9041 | 0.0183 | (0.0003, 0.4675) |
| Vit D Active | -0.5190 | 0.5951 | 0.3762 | 0.1677 | (0.2847, 1.2440) | 0.3967 | 0.1908 | (0.2735, 1.2950) |
| Age | 0.0640 | 1.0661 | 0.0295 | 0.0300 | (1.0062, 1.1296) | 0.0276 | 0.0204 | (1.0100, 1.1254) |
| Current Smoker | 0.6478 | 1.9113 | 0.5720 | 0.2574 | (0.6229, 5.8645) | 0.5095 | 0.2036 | (0.7041, 5.1884) |
| > One risk factor | 0.7410 | 2.0980 | 0.4517 | 0.1009 | (0.8656, 5.0852) | 0.5638 | 0.1887 | (0.6949, 6.3347) |
| n-3 HUFA supplementation x AfAm | 0.4077 | 1.5033 | 0.4377 | 0.3516 | (0.6375, 3.5449)* | 0.4377 | 0.3516 | (0.6724, 3.3612)* |
| n-3 HUFA supplementation x NHW | 0.0000 | 1.0000 | 0.0214 | 1.0000 | (0.9589, 1.0428)* | 0.2645 | 1.0000 | (0.5955, 1.6792)* |

**Table S4:** Regression Results after Selection of Variables by LASSO with Cardiovascular Death as the Outcome using African American and non-Hispanic White Participants after Optimal Pair Matching. The interaction terms are not selected by model. Std. Errors, p-value and 95% CI for OR are calculated using non-parametric bootstrap and parametric bootstrap. OR: odds ratio; CI: confidence interval.

| Variables | Estimate | OR | Non-parametric Bootstrap | | | Parametric Bootstrap | | |
|---|---|---|---|---|---|---|---|---|
| | | | Std. Error | P-value | 95% CI for OR | Std. Error | P-value | 95% CI for OR |
| (Intercept) | -5.6986 | 0.0034 | 1.8023 | 0.0016 | (0.0001, 0.1146) | 1.8747 | 0.0024 | (0.0001, 0.1321) |
| Female | -0.6484 | 0.5229 | 0.4225 | 0.1249 | (0.2284, 1.1969) | 0.3875 | 0.0943 | (0.2447, 1.1175) |
| Age | 0.0649 | 1.0671 | 0.0250 | 0.0094 | (1.0160, 1.1206) | 0.0250 | 0.0094 | (1.0160, 1.1206) |
| BMI | 0.0412 | 1.0421 | 0.0246 | 0.0940 | (0.9930, 1.0935) | 0.0265 | 0.1200 | (0.9893, 1.0976) |
| Current Smoker | 1.1492 | 3.1557 | 0.4270 | 0.0071 | (1.3665, 7.2872) | 0.4106 | 0.0051 | (1.4112, 7.0567) |
| Hypertension medication | 0.3457 | 1.4130 | 0.3546 | 0.3296 | (0.7052, 2.8312) | 0.3379 | 0.3063 | (0.7286, 2.7401) |
| Aspirin | 0.3741 | 1.4537 | 0.3085 | 0.2253 | (0.7941, 2.6612) | 0.2951 | 0.2049 | (0.8152, 2.5922) |

**Figure S1:** Weighted Decision Tree for Predicting the Incidence of Stroke. a. Tree for AfrAm participants after optimal pairs matching, n=3,766, weight ratio = non-diseased: diseased= 3734:32 = 117:1. b. Tree for NHW participants after optimal pair matching, n=3,766, weight ratio = non-diseased: diseased = 3743:23 = 163:1. BMI, body mass index at randomization, kg/m2; age, age at randomization to VITAL study, years; statins, baseline statin use, yes or no; sex, female or male.

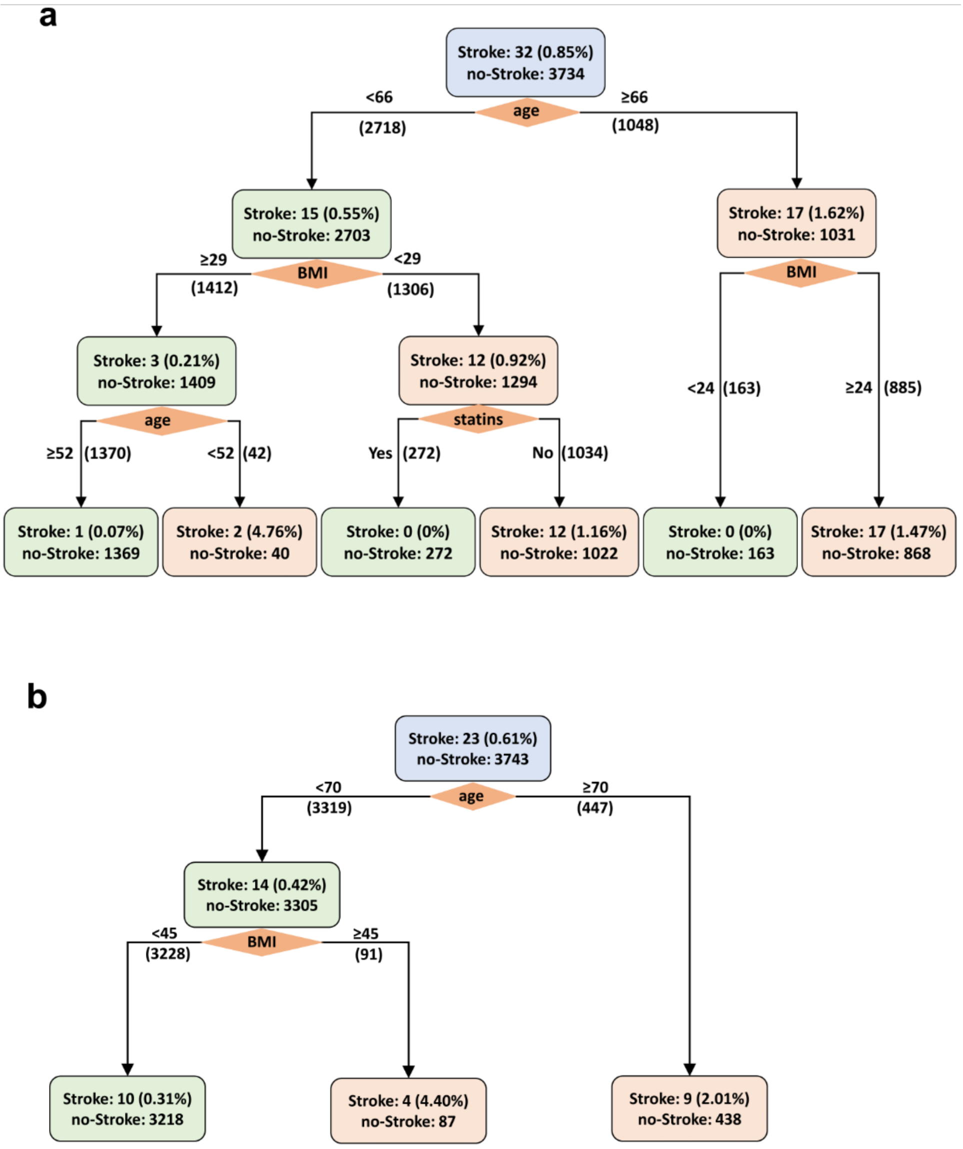

**Figure S2:** Weighted Decision Tree to Predict Cardiovascular Mortality. a. Tree for AfAm participants after optimal pairs matching, n=3,766, weight ratio = non-diseased : diseased = 3,722:44 = 84.6:1. b. Tree for NHW participants after optimal pairs matching, n=3,766, weight ratio = non-diseased : diseased = 3,732:3.0 = 109.8:1. age; Age at randomization to VITAL study, years, Aspirin; Baseline aspirin use, BMI; Body mass index at randomization, kg/m2.

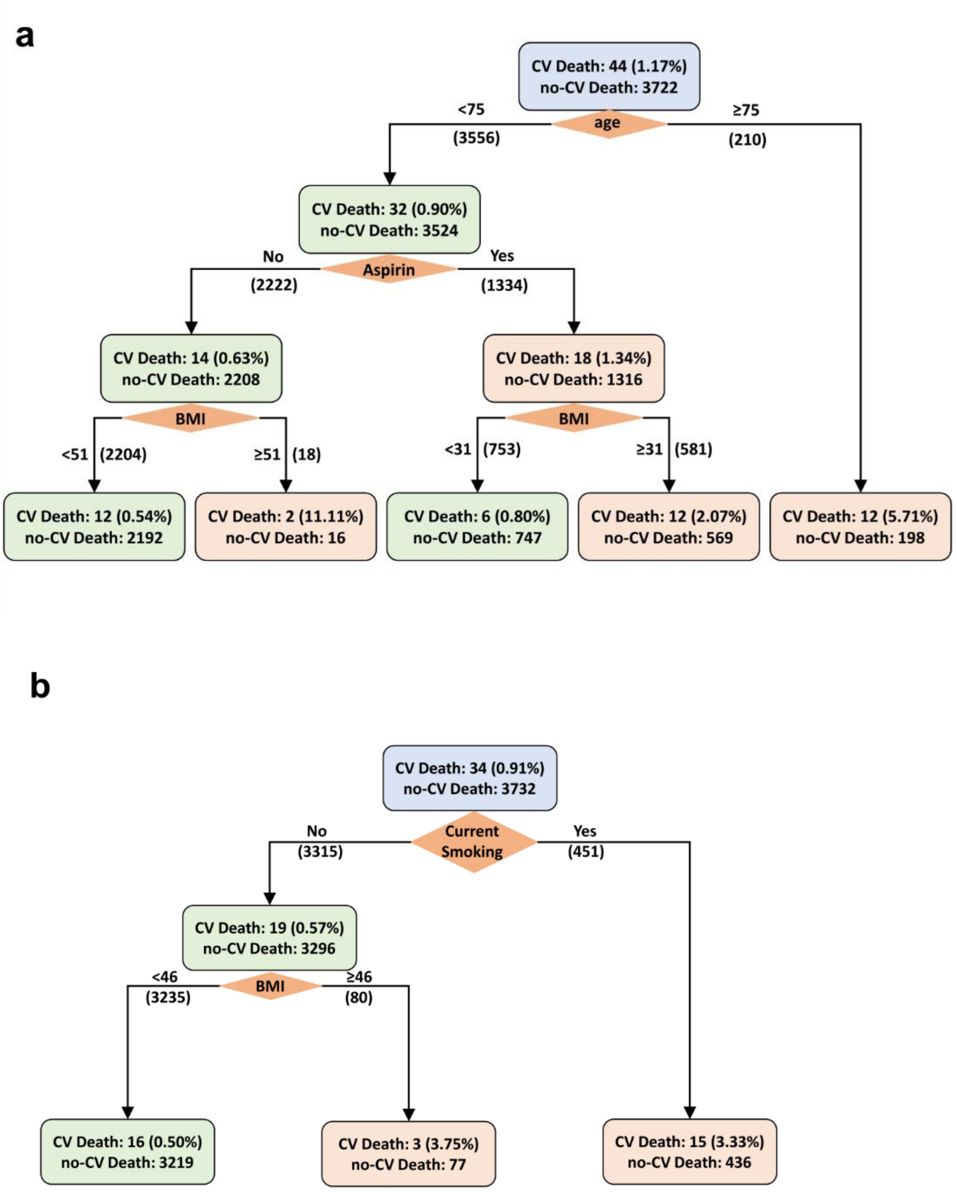

Supplement: Supplementary file 1 [file nutrients-16-02933-s001.zip › nutrients-3156905-supplementary.pdf]
